# Associations between father–mother food parenting practices and children’s dietary intake

**DOI:** 10.1017/S1368980026102638

**Published:** 2026-05-15

**Authors:** Brian K. Lo, Natalie Grafft, Mariane Helen De Oliveira, Sebastien Haneuse, In Young Park, Alejandra Cantu-Aldana, Katherine W. Bauer, Rebekah Levine Coley, Jorge E. Chavarro, Kirsten K. Davison, Jess Haines

**Affiliations:** 1 Department of Family Relations & Applied Nutrition, University of Guelph, Canada; 2 School of Social Work, Boston College, USA; 3 Department of Biostatistics, Harvard University T H Chan School of Public Health, USA; 4 Department of Social Welfare, Chungnam National University, Republic of Korea; 5 Division of Human Genetics, The University of Texas Rio Grande Valley, USA; 6 Department of Nutritional Sciences, University of Michigan School of Public Health, USA; 7 Lynch School of Education and Human Development, Boston College, USA; 8 Department of Nutrition and Epidemiology, Harvard University T H Chan School of Public Health, USA

**Keywords:** Fathers, Food parenting, Child diet, Dyadic analysis

## Abstract

**Objective:**

To examine whether the number of parents using high levels of structure or coercive control food parenting practices is associated with children’s dietary intake.

**Design:**

Cross-sectional analysis of father–mother dyads from the Fathers & Families cohort study. Fathers and mothers independently reported their food parenting practices and child’s dietary intake. Multivariate logistic regression models estimated associations between the number of parents (0, 1 or 2) using high structure or coercive control practices and children’s intake of fruit, vegetables, fast food and sugar-sweetened beverages (SSB), adjusting for demographics and recruitment site.

**Setting:**

United States

**Participants:**

474 father–mother dyads with a child aged 2–6 years

**Results:**

Structure, but not coercive control, was positively associated with vegetable intake. Compared with none, having one parent report high structure increased the odds of children consuming vegetables > once/day (adjusted OR (aOR) = 2·09; 95 % CI: (1·06, 4·54)); associations were stronger when both parents reported high structure. Structure was also associated with lower fast food and SSB intake frequency. Compared with none, having one parent report high structure increased the odds of children consuming fast food < once/week (aOR = 1·79; 95 % CI: (1·23, 2·62)) and limiting SSB to < once/week (aOR = 1·52; 95 % CI: (1·03, 2·23)); associations were stronger when both parents reported high structure. Compared with none, high coercive control by one (aOR = 0·69; 95 % CI: (0·49, 0·96)) or both parents (aOR = 0·57; 95 % CI: (0·33, 1·00)) was associated with lower odds of limiting SSB to < once/week.

**Conclusions:**

Children’s dietary patterns were healthiest when both parents used structure-based food parenting practices. Coercive control from one or both parents was associated with greater SSB intake frequency.

Parents are key agents in childhood obesity prevention. Their use of specific food parenting practices has been linked to children’s intake of fruit, vegetables, fast food and sugar-sweetened beverages (SSB)^(1–4)^, which are critical dietary targets in early childhood obesity prevention^(5,6)^. Food parenting practices are often conceptualised along dimensions of structure and coercive control. Structure-based practices involve parents creating a consistent and supportive eating environment that facilitates children’s development of healthy eating habits^(7)^, including providing consistent mealtime environments, monitoring, modelling and creating healthy food environments^(7–9)^. In contrast, coercive control-based practices involve parents prioritising feeding goals and preferences while overlooking children’s emotional and psychological needs^(7)^, such as pressuring, restricting or using food to regulate emotions or as rewards^(7–9)^. Structure-based practices are generally associated with healthier dietary intake, whereas coercive control-based practices are associated with less healthy dietary patterns^(1–3,10,11)^.

Despite Family Systems Theory emphasising the interdependence of family members^(12)^, and emerging evidence suggesting that fathers and mothers may differ in their food parenting practices^(13,14)^, most studies examining food parenting have focused almost exclusively on mothers, rarely considering the food parenting practices used by both fathers and mothers within the same household. One systematic review of forty-four studies on restrictive feeding practices found that fourteen included only mothers, and among the thirty that included fathers and mothers, mothers comprised at least 80 % of participants in most cases^(15)^. Similarly, a second systematic review reported that most studies using parent-report measures disproportionately included mothers, although the exact proportion was not reported^(1)^.

Overlooking the joint influence of fathers’ and mothers’ food parenting practices within the same household may limit the development of effective family-centred obesity prevention strategies, particularly those designed to promote shared, health-supportive parenting. To address this gap, this study assessed whether, within households comprising a father and a mother, the number of parents using high levels of structure or coercive control food parenting practices was associated with children’s intake of fruit, vegetables, fast food and SSB, as reported by parents. We hypothesised that children with more parents using high-structure practices would consume fruit and vegetables more frequently and fast food and SSB less frequently, whereas those with more parents using high-coercive-control practices would exhibit the opposite pattern of intake.

## Methods

### Sample

This study included fathers and their female co-parents from Fathers & Families (F&F), a prospective cohort study on fathers’ roles in promoting child health, described in detail elsewhere^(16)^. The F&F cohort includes 750 fathers recruited from the Growing Up Today Study (GUTS)^(17)^ between 2021 and 2022, and 522 fathers recruited from Michigan Medicine between 2022 and 2023 (*n* 1272). Eligible fathers were biological, adoptive or social fathers of a child aged 1–6 years with whom they lived at least 50 % of the time. Fathers were invited to recruit co-residential co-parents; 582 co-parents enrolled. Based on our research aim, we restricted the sample to father–mother dyads to examine food parenting practices within households with both male and female caregivers. Fathers with a non-female co-parent (*n* 8) were therefore excluded. Dyads with a child younger than 2 years (*n* 93) were also excluded, as our food parenting measure was designed for children aged 2 years and above^(16,18)^. This resulted in a total possible sample of 481 father–mother dyads. Missing data in the father–mother dyad sample were low (< 1 % for fathers and ≤ 2 % for mothers at the item level). Dyads with missing data (*n* 7) did not differ in demographics, food parenting practices or child diets and were excluded, yielding a final analytic sample of 474 father–mother dyads. This study was approved by the Mass General Brigham Institutional Review Board (protocol #: 2020P002688) and Boston College Institutional Review Board (protocol #: 22.201.01).

### Measures

#### Dyadic food parenting practices

Fathers and mothers independently reported their food parenting practices using a brief version of the Comprehensive Feeding Practices Questionnaire (CFPQ)^(18)^: five items assessed structure-based practices (two monitoring, two environment and one modelling) and four assessed coercive control-based practices (two emotional regulation and two food as reward) (online Supplementary Figure 1)^(7)^. A brief CFPQ (rather than the lengthy forty-nine-item full CFPQ) was used in response to evidence that fathers are more likely to participate in research when respondent burden is minimised^(19)^. This brief version underwent rigorous development and validation within the F&F sample, including theory- and expert-informed item selection, and demonstrated factorial validity, measurement invariance across fathers and mothers, internal reliability and concurrent validity, as reported elsewhere^(16)^. Each item was rated on a four-point Likert scale (1 = ‘strongly disagree’ to 4 = ‘strongly agree’); structure and coercive control subscale scores were calculated as weighted means based on factor loadings to reflect each item’s contribution to the construct (online Supplementary Figure 1). Parents were dichotomised as ‘high’ or ‘low’ on each subscale using a threshold of ≥ 3, identifying those who, on average, agreed or strongly agreed as high users of the respective practice. Two categorical variables were created to indicate whether 0, 1 or 2 parents in the same household reported high structure and coercive control.

#### Child dietary outcomes

Parents reported their child’s diet using five validated items from the National Health and Nutrition Examination Survey (NHANES) Dietary Screener Questionnaire (DSQ)^(20,21)^. A brief dietary frequency screener was used to minimise survey burden. Specifically, parents reported how often, on average, over the past 30 days their child consumed: (a) fruit (fresh, frozen or canned); (b) vegetables (raw, cooked, canned or frozen); (c) regular soda or pop (excluding diet); (d) sweetened fruit, sports or energy drinks (excluding diet); and (e) food from a fast-food restaurant (e.g. McDonald’s, Burger King and Taco Bell). Response options were ‘never’, ‘less than once per week’, ‘once per week’, ‘2–4 times per week’, ‘nearly daily or daily’, ‘2–4 times per day’ and ‘5 or more times per day.’ Parents were instructed to consider all meals and snacks consumed at home, school/daycare, restaurants or elsewhere. All responses were first converted to weekly frequencies. Daily frequencies were multiplied by 7, and ranged responses (e.g. 2–4 times/d) were coded to the midpoint. ‘Never’ was coded as 0, ‘less than once per week’ as 0·5 and ‘5 or more times per day’ as 35 times per week. Soda and sweet drink frequencies were summed to calculate total weekly SSB intake. Weekly frequencies were then converted to daily frequencies by dividing by 7, consistent with National Cancer Institute guidance^(22)^. Based on dietary guidelines recommending that children consume a variety of fruits and vegetables throughout the day and limit added sugar, saturated fat and Na^(23)^, children’s diets were coded into four binary variables indicating whether the child consumed (1) fruit > once/day, (2) vegetables > once/day, (3) fast food < once/week and (4) SSB < once/week.

In 89 % of dyads, mothers completed the survey within 30 days of fathers’ responses, and in 70 %, within 14 days. Prior studies suggest that parents’ perceptions of their child’s weight status and social desirability bias may contribute to over-reporting of healthy foods and under-reporting of unhealthy foods^(24–28)^. To address this, we used the lower parent-reported value for fruit and vegetables and the higher report for fast food and SSB. For the eight dyads (father *n* 1; mother *n* 7) where only one parent reported, that parent’s responses were used. Fathers’ and mothers’ reports showed reasonable concordance (online Supplementary Table S1).

#### Demographics

Fathers reported their race/ethnicity and education and their child’s age and sex. Mothers reported their own race/ethnicity. Mother’s education was reported by fathers among those recruited from Michigan Medicine and self-reported by mothers in GUTS.

### Statistical analyses

Descriptive statistics were used to characterise participants’ weighted demographics and children’s intake of fruit, vegetables, fast food and SSB.

Four multivariate logistic regression models were fit to estimate the associations between fathers’ and mothers’ food parenting practices, operationalised as the number of parents using high structure or coercive control (0, 1 or 2), and each binary child diet outcome (fruit, vegetables, fast food and SSB), reported as OR. The two food-parenting variables representing father–mother dyadic reports of structure and coercive control were entered simultaneously in each model. Both unadjusted and adjusted models were fit, with the latter including fathers’ education, mothers’ education (different from fathers or not), fathers’ race/ethnicity, mothers’ race/ethnicity (different from fathers or not), child’s age, child’s sex and recruitment site. A two-degree-of-freedom Wald chi-squared test assessed the joint significance of each food parenting practice variable.

To address the risk of selection bias in the father–mother dyad sample, all analyses (descriptive statistics and regression models) were weighted using inverse probability weighting^(29)^. Weights were calculated as the inverse of the predicted probability of membership into the observed father–mother dyad sample based on demographic (i.e. recruitment site, father’s race/ethnicity, father’s age, child’s age, number of children in the household and household income) and parenting characteristics (co-parenting relationship quality and father responsibility) that were hypothesised to influence mothers’ enrolment in F&F. Characteristics of the full sample of fathers with a female co-parent and a child aged 2 years and above (*n* 1003) and of the dyad sample (*n* 481) are shown in online Supplementary Table S2.

## Results

Most fathers (67·9 %) and mothers (72·1 %) were non-Hispanic White; in nearly one-quarter of dyads, fathers and mothers reported different race/ethnicity (Table 1). Most fathers (86·5 %) and mothers (87·7 %) held a bachelor’s degree or higher, with 44·8 % reporting different levels of educational attainment. Just over half of the children were male (51·0 %), and most were aged 3–5 years (59·4 %).

**Table 1.** Weighted demographic characteristics of 474 father–mother dyads Table 1 long description.

	Father–mother dyads *n* 474		
	Weighted %*	Mean*	sd *
Father’s race/ethnicity			
Hispanic/Latino	5·3		
Non-Hispanic Asian	13·9		
Non-Hispanic Black	7·4		
Non-Hispanic White	67·9		
Non-Hispanic multi-racial	4·1		
Non-Hispanic other	1·4		
Father’s education			
Some college or less	13·5		
Bachelor’s degree	33·9		
Postgraduate degree	52·6		
Mother’s race/ethnicity			
Hispanic/Latino	5·2		
Non-Hispanic Asian	13·9		
Non-Hispanic Black	4·7		
Non-Hispanic White	72·1		
Non-Hispanic multi-racial	3·0		
Non-Hispanic other	1·2		
Mother’s education			
Some college or less	12·3		
Bachelor’s degree	34·1		
Postgraduate degree	53·6		
Mother’s race/ethnicity different than father’s race/ethnicity			
Yes	23·7		
Mother’s education different than father’s education			
Yes	44·8		
Child age in months		45·6	15·5
Child sex			
Male	51·0		
Female	49·0		
Recruitment site			
GUTS	59·6		
Michigan Medicine	40·4		
Child diets			
Fruit > once/day	40·9		
Vegetables > once/day	15·7		
Fast food < once/week	59·9		
Sugar-sweetened beverages < once/week	62·5		

GUTS, Growing Up Today Study.

* Applied inverse probability weighting.

Participant demographics by number of parents using high structure and coercive control are presented in online Supplementary Table S3. Of the 224 dyads in which only one parent reported high structure, 216 were mothers and 8 were fathers. Similarly, among the 112 dyads where only one parent reported high coercive control, 78 were mothers and 34 were fathers.

### Fruit intake

There were no associations between the number of parents using high structure or coercive control and children’s fruit intake (Table 2; unadjusted results in online Supplementary Table S4).

**Table 2.** Adjusted OR for the associations between father–mother dyadic food parenting practices and children’s dietary outcomes (*n* 474 father–mother dyads) Table 2 long description.

	Consuming fruit more than once per day		Consuming vegetables more than once per day		Consuming fast food less than once per week		Consuming sugar-sweetened beverages less than once per week	
	Adjusted OR^†,‡^	95 % CI^†,‡^	Adjusted OR^†,‡^	95 % CI^†,‡^	Adjusted OR^†,‡^	95 % CI^†,‡^	Adjusted OR^†,‡^	95 % CI^†,‡^
# of parents reported high structure^§^								
0	Ref.		**Ref.** ^***^		**Ref.** ^***^		**Ref.** ^***^	
1	1·04	0·70, 1·55	**2·09**	**1·06, 4·54**	**1·79**	**1·23, 2·62**	**1·52**	**1·03, 2·23**
2	1·33	0·89, 2·01	**4·80**	**2·47, 10·32**	**3·43**	**2·83, 5·17**	**2·75**	**1·82, 4·17**
# of parents reported high coercive control^||^								
0	Ref.		Ref.		Ref.		Ref.^*^	
1	0·85	0·61, 1·18	0·63	0·38, 1·00	0·79	0·57, 1·10	**0·69**	**0·49, 0·96**
2	0·50	0·27, 0·89	0·71	0·27, 1·57	0·60	0·35, 1·03	**0·57**	**0·33, 1·00**

The bolded numbers in the table reflect the 2-degree-of-freedom Wald test for the joint significance of each food parenting practice variable (*P < 0·05, **P < 0·01, ***P < 0·001) and do not refer to the significance levels of the individual coefficients.

† Controlled for fathers’ education, mothers’ education (different from fathers or not), fathers’ race/ethnicity, mothers’ race/ethnicity (different from fathers or not), child’s age, child’s sex and recruitment site.

‡ Applied inverse probability weighting.

§ Number of parents reported high structure: 0 = 79 dyads, 1 = 224 dyads and 2 = 171 dyads.

|| Number of parents reported high coercive control: 0 = 329 dyads, 1 = 112 dyads and 2 = 33 dyads.

### Vegetable intake

Structure, but not coercive control, was associated with children’s vegetable intake. Compared with no parents reporting high structure, one parent reporting high structure was associated with greater odds of children consuming vegetables > once/day (adjusted OR (aOR) = 2·09; 95 % CI: (1·06, 4·54)), with even greater odds when both parents reported high structure (aOR = 4·80; 95 % CI: (2·47, 10·32)). Coercive control was not associated with children’s vegetable intake.

### Fast food intake

Compared with no parents reporting high structure, one parent reporting high structure was associated with greater odds of children consuming fast food < once/week (aOR = 1·79; 95 % CI: (1·23, 2·62)), with even higher odds when both parents reported high structure (aOR = 3·43; 95 % CI: (2·83, 5·17)). Coercive control was not associated with children’s fast food intake.

### Sugar-sweetened beverage intake

Compared with no parents reporting high structure, one parent reporting high structure was associated with greater odds of children consuming SSB < once/week (aOR = 1·52; 95 % CI: (1·03, 2·23)), with even higher odds when both parents reported high structure (aOR = 2·75; 95 % CI: (1·82, 4·17)). In contrast, compared with no parents reporting high coercive control, having one parent report high coercive control was associated with lower odds of children consuming SSB < once/week (aOR = 0·69; 95 % CI: (0·49, 0·96)), with a similar trend when both parents reported high coercive control (aOR = 0·57; 95 % CI: (0·33, 1·00)).

## Discussion

In this study, we found consistent evidence that a greater number of parents reporting high structure was associated with certain healthier dietary patterns among children, including higher odds of consuming vegetables > once/day and consuming fast foods and SSB < once/week. These results highlight the importance of considering whether one or both parents use a given food parenting practice, as well as the type of practice (structure *v*. coercive control), to better understand parents’ roles in shaping children’s diets.

A graded pattern of associations was observed for structure. As the number of parents reporting high structure increased, so did the odds of children consuming vegetables > once/day and limiting fast food and SSB intake to < once/week. Children were about two to five times more likely to meet these targets when one or both parents (*v*. none) reported high structure. These results align with recent systematic reviews showing that structure-based practices, such as modelling and regulating food availability, are consistently associated with children’s diets^(1,3,4)^. Our findings suggest that the benefits of high structure are amplified when both parents are involved, echoing Family Systems Theory’s emphasis on the joint influence of parents on children’s outcomes^(12)^. Findings also highlight the importance of parents adopting consistent approaches to reinforce messages and provide a cohesive feeding environment. The absence of an association between high structure and fruit intake may indicate that other unmeasured practices, such as autonomy support through education and encouragement, play a greater role in shaping fruit consumption, as seen in prior studies^(1,10,30)^.

These results have implications for childhood obesity prevention. Although many interventions target one caregiver, often mothers^(31,32)^, our findings suggest that engaging both fathers and mothers in structure-based food parenting may yield greater improvements in children’s diets. As fathers are increasingly involved in food parenting^(33,34)^ and express interest in health programmes^(35)^, interventions should support both parents in adopting consistent structure-based practices to reduce discretionary food intake and promote healthy eating and should encourage alignment of their food parenting practices to minimise conflict and inconsistency in child feeding.

In contrast, the associations between high coercive control and children’s diets were weaker and less consistent. Children were 30 to 40 % less likely to limit SSB intake when one or both parents reported high coercive control. No significant association was found for fruit, vegetable or fast food intake. Although the general lack of associations between coercive control-based practices and children’s diets contradicted our hypothesis, it is consistent with the variability documented in recent systematic reviews, which found that coercive control-based practices are generally less consistently associated with children’s diets than structure-based practices^(1,4,10,15)^. The associations observed for SSB intake, but not for other foods, also align with evidence suggesting that links between coercive control-based practices and child diet may be food-specific. For example, using food as a reward (i.e. offering specific foods to reinforce desired behaviours) is more often associated with unhealthy items such as SSB than with fruits and vegetables^(1,36)^. Although both SSB and fast food are considered unhealthy, SSB are less expensive, widely available and easily consumed at home and in small quantities, making them more likely to be used within coercive control practices as food rewards. Using SSB to manage behaviour may increase their perceived value to children, potentially promoting greater consumption^(37)^.

This study is the first to concurrently examine father and mother food parenting practices within families using a large sample; however, several limitations should be noted. First, the cross-sectional design precludes causal inference. Second, restricting the sample to father–mother dyads with children aged 2 to 6 years limits generalisability to other caregiver constellations, including same-sex caregivers, and to other child age groups. Third, reliance on parent-reported child diet may introduce recall and social desirability bias, although our use of the more conservative report may have mitigated this. Fourth, sample characteristics limited our ability to examine differential dyadic patterns (e.g. father high *v*. mother low), as mothers more often reported high structure or coercive control. Fifth, our findings reflect only two higher-order domains of food parenting, namely structure and coercive control, as measured using a selected subset of CFPQ items. Some important CFPQ items, such as those related to pressure to eat and restriction, were not included in the brief CFPQ, despite prior evidence linking them to children’s dietary outcomes^(1,10,11)^. Sixth, none of the CFPQ items directly correspond to the specific dietary outcomes assessed in this study, which may have also contributed to the absence of some expected associations. Lastly, we used a brief dietary screener that assessed intake frequency only, without capturing portion size or dietary variety; therefore, these measures do not represent a comprehensive assessment of diet quality.

## Conclusions

Children’s vegetable, fast food and SSB consumption patterns were most favourable when both parents used structure-based food parenting practices. Coercive control was associated with more frequent SSB intake but showed no evidence of stronger associations when practiced by both parents.

## Supporting information

10.1017/S1368980026102638.sm001 Lo et al. supplementary material 1 Lo et al. supplementary material

10.1017/S1368980026102638.sm002 Lo et al. supplementary material 2 Lo et al. supplementary material

10.1017/S1368980026102638.sm003 Lo et al. supplementary material 3 Lo et al. supplementary material

10.1017/S1368980026102638.sm004 Lo et al. supplementary material 4 Lo et al. supplementary material

10.1017/S1368980026102638.sm005 Lo et al. supplementary material 5 Lo et al. supplementary material

## Supplementary material

For supplementary material accompanying this paper, visit https://doi.org/10.1017/S1368980026102638

## Table 1. Long description

The table presents the weighted demographic characteristics of 474 father-mother dyads. It includes data on the race/ethnicity and education levels of both fathers and mothers, as well as the age and sex of their children. The table has 39 rows and 4 columns. The columns are labeled ‘Father-mother dyads n 474’, ‘Weighted %’, ‘Mean’, and ‘SD’. The rows detail the race/ethnicity and education of fathers and mothers, whether the mother’s race/ethnicity or education differs from the father’s, child age in months, child sex, recruitment site, and child diets. Notable trends include that most fathers (67.9%) and mothers (72.1%) are non-Hispanic White, and nearly one-quarter of dyads have parents of different race/ethnicity. Most fathers (86.5%) and mothers (87.7%) hold a bachelor’;s degree or higher, with 44.8% reporting different levels of educational attainment. Just over half of the children are male (51.0%), and most are aged 3-5 years (59.4%).

Navigate back to Table 1..

## Table 2. Long description

The table presents adjusted odds ratios for the associations between father-mother dyadic food parenting practices and children’s dietary outcomes, based on data from 474 father-mother dyads. The table includes columns for different dietary outcomes such as consuming fruit more than once per day, consuming vegetables more than once per day, consuming fast food less than once per week, and consuming sugar-sweetened beverages less than once per week. Each column provides adjusted odds ratios and 95 percent confidence intervals for different numbers of parents reported using high structure and high coercive control. Notable trends include varying odds ratios depending on the number of parents using high structure or coercive control, with specific values provided for each dietary outcome.

Navigate back to Table 2..
