# Supplementary material for: Associations between father–mother food parenting practices and children’s dietary intake
Source: Public Health Nutr. 2026 May 15;29(1):e116. doi: 10.1017/S1368980026102638 (PMC13434556; doi:10.1017/S1368980026102638)
Supplement: Supplementary file 1 [file S1368980026102638sup005.pdf]

Supplementary Figure 1: Factor loadings for structure and coercive control among fathers and mothers

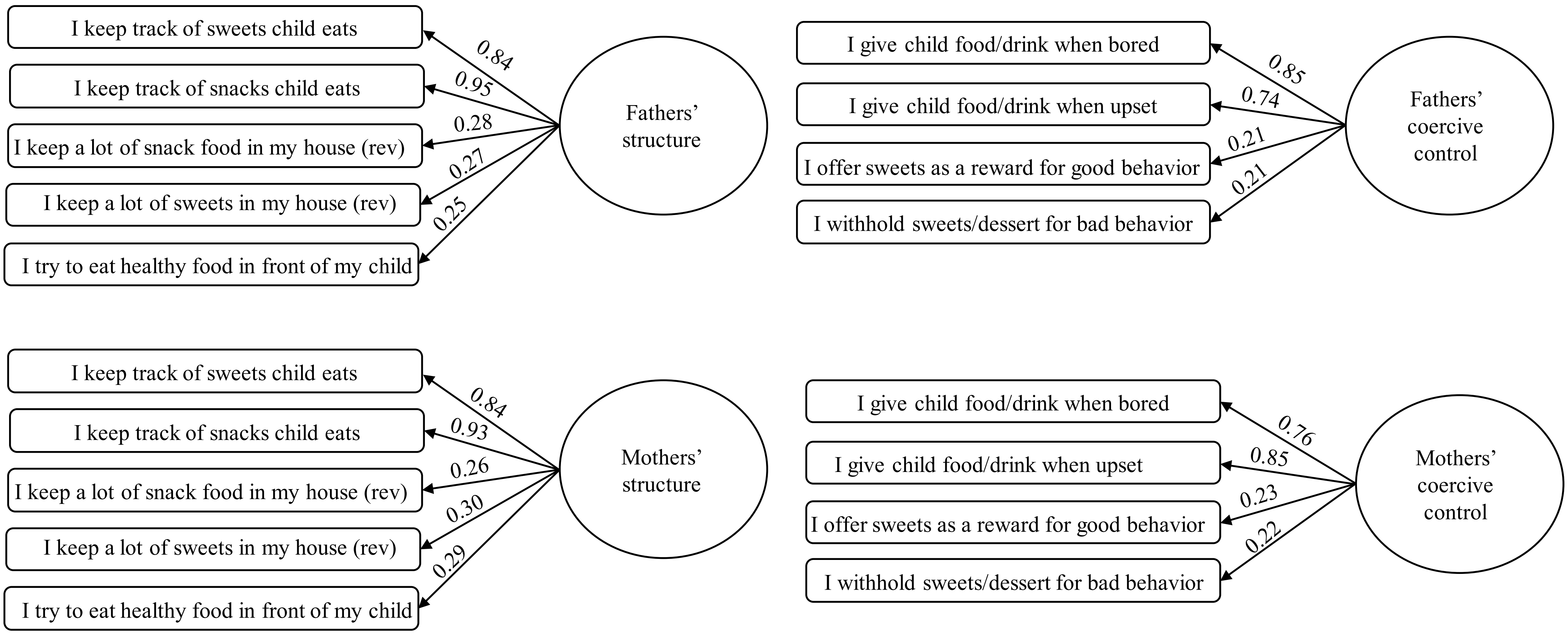

rev: reverse coded item
