# Supplementary material for: Associations between father–mother food parenting practices and children’s dietary intake
Source: Public Health Nutr. 2026 May 15;29(1):e116. doi: 10.1017/S1368980026102638 (PMC13434556; doi:10.1017/S1368980026102638)
Supplement: Supplementary file 2 [file S1368980026102638sup001.docx]

**Supplementary Table S1.** Fathers’ and Mothers’ Concordance of Reporting Child Dietary Outcomes

| **Child dietary outcomes** | **Parental reporting concordance**^a^ |
| --- | --- |
|  | Weighted %^b^ |
| Fruit | 69.6 |
| Vegetables | 73.9 |
| Fast Food | 64.9 |
| Sugar-sweetened Beverages | 75.2 |

*Note:* ^a^Concordance was calculated based on the dichotomized diet variable (e.g., parents agree or disagree if their child consumes fruit >once/day). ^b^Applied inverse probability weighting.
