# Supplementary material for: Associations between father–mother food parenting practices and children’s dietary intake
Source: Public Health Nutr. 2026 May 15;29(1):e116. doi: 10.1017/S1368980026102638 (PMC13434556; doi:10.1017/S1368980026102638)
Supplement: Supplementary file 3 [file S1368980026102638sup002.docx]

**Supplementary Table S2.** Demographic Characteristics of Fathers & Families Participants

|  | **Total sample of fathers with a female co-parent and child ≥2 years**  (*n* = 1,003) | **Father-mother dyad sample with child ≥2 years**  (*n* = 481) |
| --- | --- | --- |
| **Demographic Characteristics** | | |
| **Father education, %** |  |  |
| Some college or less | 14.7 | 14.3 |
| Bachelor’s degree | 36.6 | 33.1 |
| Postgraduate degree | 48.3 | 52.2 |
| Missing | <1.0 | <1.0 |
| **Father race/ethnicity, %** |  |  |
| Hispanic/Latino | 5.7 | 6.2 |
| Non-Hispanic Asian | 13.4 | 16.2 |
| Non-Hispanic Black | 7.5 | 7.3 |
| Non-Hispanic White | 67.1 | 63.2 |
| Non-Hispanic multi-racial | 4.3 | 5.2 |
| Non-Hispanic other^a^ | 1.5 | 1.7 |
| Missing | <1.0 | <1.0 |
| **Father age (years), mean (SD)** | 36.6 (4.0) | 36.5 (4.2) |
| **Household income (USD in thousands), mean (SD)** | 166.3 (91.4) | 163.4 (91.5) |
| **Number of children in household, mean (SD)** | 2.0 (0.95) | 2.0 (0.91) |
| **Child age (months), mean (SD)** | 45.8 (15.7) | 45.4 (15.7) |
| **Recruitment site, %** |  |  |
| GUTS | 59.7 | 49.3 |
| Michigan Medicine | 40.3 | 50.7 |
| **Parenting Characteristics** | | |
| Father responsibility,^b^ mean (SD) | 2.7 (0.5) | 2.7 (0.5) |
| Co-parent relationship quality,^c^ mean (SD) | 2.6 (0.4) | 2.7 (0.3) |
| *Note:* fathers reported on all items; ^a^includes fathers who identify as American Indian or Alaskan Native. ^b^Mean score of 9 items adapted from the parental responsibility scale (from McBride BAM, G. A comparison of mother and father involvement with their preschool age children. *Early Child Res Q*. 1993;8(4):457-477.). ^c^Mean score of 12 items adapted from the coparenting relationship scale (from Feinberg ME, Brown LD, Kan ML. A Multi-Domain Self-Report Measure of Coparenting. *Parent Sci Pract*. 2012;12(1):1-21.) | | |
