# Supplementary material for: Associations between father–mother food parenting practices and children’s dietary intake
Source: Public Health Nutr. 2026 May 15;29(1):e116. doi: 10.1017/S1368980026102638 (PMC13434556; doi:10.1017/S1368980026102638)
Supplement: Supplementary file 4 [file S1368980026102638sup003.docx]

**Supplementary Table S3.** Weighted Demographic Characteristics of Father-Mother Dyads by Number of Parents Reported High Structure or Coercive Control Food Parenting Practices (*N* = 474)

|  | **Number of parents reported high structure in each dyad** | | | **Number of parents reported high coercive control in each dyad** | | |
| --- | --- | --- | --- | --- | --- | --- |
|  | **0**  **(n = 79)** | **1**  **(n = 224)** | **2**  **(n = 171)** | **0**  **(n = 329)** | **1**  **(n = 112)** | **2**  **(n = 33)** |
|  | **16.7%** | **47.3%** | **36.0%** | **69.4%** | **23.6%** | **7.0%** |
|  | **Weighted %**^a^ | | | **Weighted %**^a^ | | |
| **Father race/ethnicity** |  |  |  |  |  |  |
| Hispanic/Latino | 5.5 | 4.2 | 6.7 | 5.4 | 6.1 | 2.4 |
| Non-Hispanic Asian | 13.8 | 15.7 | 11.5 | 10.8 | 19.6 | 25.5 |
| Non-Hispanic Black | 9.2 | 7.1 | 6.8 | 7.9 | 6.3 | 5.9 |
| Non-Hispanic White | 66.3 | 66.5 | 70.3 | 70.5 | 61.3 | 63.8 |
| Non-Hispanic multi-racial | 3.6 | 4.6 | 3.9 | 3.9 | 6.0 | 0.0 |
| Non-Hispanic other | 1.6 | 1.9 | <1.0 | 1.6 | <1.0 | 2.4 |
| **Father education** |  |  |  |  |  |  |
| Some college or less | 24.4 | 13.8 | 8.0 | 11.7 | 17.8 | 16.5 |
| Bachelor’s degree | 34.1 | 32.4 | 35.7 | 38.4 | 22.6 | 27.2 |
| Postgraduate degree | 41.5 | 53.8 | 56.4 | 49.9 | 59.6 | 56.3 |
| **Mother race/ethnicity** |  |  |  |  |  |  |
| Hispanic/Latino | 4.0 | 5.8 | 5.0 | 6.2 | 3.7 | 0.0 |
| Non-Hispanic Asian | 12.9 | 16.0 | 11.7 | 10.9 | 20.6 | 21.2 |
| Non-Hispanic Black | 5.5 | 4.6 | 4.6 | 4.7 | 5.3 | 3.6 |
| Non-Hispanic White | 71.8 | 69.6 | 75.2 | 73.7 | 66.5 | 73.0 |
| Non-Hispanic multi-racial | 5.8 | 1.9 | 3.0 | 3.3 | 2.1 | 2.3 |
| Non-Hispanic other | 0.0 | 2.2 | <1.0 | 1.1 | 1.8 | 0.0 |
| **Mother education** |  |  |  |  |  |  |
| Some college or less | 22.6 | 13.2 | 6.5 | 11.2 | 14.0 | 18.2 |
| Bachelor’s degree | 31.5 | 35.8 | 33.2 | 34.1 | 36.5 | 26.8 |
| Postgraduate degree | 45.9 | 51.0 | 60.3 | 54.8 | 49.5 | 55.0 |
| **Mother race/ethnicity different than father’s race/ethnicity** |  |  |  |  |  |  |
| Yes | 26.1 | 23.7 | 22.5 | 24.5 | 25.0 | 11.4 |
| **Mother education different than father’s education** |  |  |  |  |  |  |
| Yes | 50.3 | 44.5 | 42.7 | 46.1 | 44.3 | 34.6 |
| **Child age** |  |  |  |  |  |  |
| 2 years | 23.9 | 31.9 | 37.2 | 36.7 | 22.7 | 22.9 |
| 3-5 years | 67.1 | 59.8 | 55.4 | 54.9 | 68.5 | 74.4 |
| 6+ years | 9.0 | 8.3 | 7.4 | 8.4 | 8.8 | 2.7 |
| **Child sex** |  |  |  |  |  |  |
| Male | 53.3 | 52.8 | 47.6 | 50.4 | 49.5 | 62.0 |
| **Recruitment site** |  |  |  |  |  |  |
| GUTS | 57.1 | 58.4 | 62.4 | 61.3 | 54.1 | 61.4 |
| Michigan Medicine | 42.9 | 41.6 | 37.6 | 38.7 | 45.9 | 38.6 |
| **Child diets** |  |  |  |  |  |  |
| Fruit >once/day | 33.4 | 38.7 | 47.1 | 43.3 | 37.8 | 27.1 |
| Vegetables >once/day | 5.8 | 12.0 | 25.1 | 17.7 | 11.5 | 9.8 |
| Fast food <once/week | 40.2 | 56.8 | 73.2 | 63.2 | 54.7 | 44.8 |
| Sugar-sweetened beverages <once/week | 45.6 | 59.6 | 74.2 | 66.9 | 53.6 | 48.6 |

*Note.* ^a^Applied inverse probability weighting.
