# Supplementary material for: Associations between father–mother food parenting practices and children’s dietary intake
Source: Public Health Nutr. 2026 May 15;29(1):e116. doi: 10.1017/S1368980026102638 (PMC13434556; doi:10.1017/S1368980026102638)
Supplement: Supplementary file 5 [file S1368980026102638sup004.docx]

**Supplementary Table S4.** Unadjusted Odds Ratios for Associations between Father-Mother Food Parenting Practices and Child Dietary Outcomes (*N* = 474)

|  | **Consuming fruit more than once per day** | **Consuming vegetables more than once per day** | **Consuming fast food less than once per week** | **Consuming sugar-sweetened beverages less than once per week** |
| --- | --- | --- | --- | --- |
|  | Unadjusted OR  (95% CI)^a^ | Unadjusted OR  (95% CI)^a^ | Unadjusted OR  (95% CI)^a^ | Unadjusted OR  (95% CI)^a^ |
| **# of parents reported high structure**^b^ |  |  |  |  |
| 0 | **Ref.*** | **Ref.***** | **Ref.***** | **Ref.***** |
| 1 | **1.21**  **(0.83, 1.78)** | **2.21**  **(1.13, 4.76)** | **1.92**  **(1.33, 2.77)** | **1.75**  **(1.22, 2.51)** |
| 2 | **1.65**  **(1.25, 2.45)** | **5.17**  **(2.70, 11.02)** | **3.81**  **(2.59, 5.67)** | **3.19**  **(2.16, 4.74)** |
| **# of parents reported high coercive control**^c^ |  |  |  |  |
| 0 | Ref. | Ref. | Ref. | **Ref.**** |
| 1 | 0.84  (0.61, 1.14) | 0.69  (0.43, 1.08) | 0.77  (0.56, 1.05) | **0.61**  **(0.45, 0.84)** |
| 2 | 0.54  (0.30, 0.93) | 0.69  (0.27, 1.50) | 0.61  (0.36, 1.07) | **0.59**  **(0.35, 0.98)** |

*Note.* CI: confidence interval; OR: odds ratio. The bolded numbers in the table reflect the 2-degree of freedom Wald test for the joint significance of each food parenting practice variable (**p* < 0.05, ***p* < 0.01, ****p* < 0.001) and do not refer to the significance levels of the individual coefficients. ^a^Applied inverse probability weighting. ^b^Number of parents reported high structure: 0 = 79 dyads, 1 = 224 dyads, 2 = 171 dyads. ^c^Number of parents reported high coercive control: 0 = 329 dyads, 1 = 112 dyads, 2 = 33 dyads.
